# Places Visited, Maintained, or Abandoned Outside Home: A Community Mobility Need of Older Adults With Dementia

**DOI:** 10.1093/geroni/igaa057.2338

**Published:** 2020-12-16

**Authors:** Isabel Margot-Cattin, Sophie Gaber, Nicolas Kuhne, Camilla Malinowski, Louise Nygard

**Affiliations:** 1 University of Applied Sciences and Arts of Western Switzerland (HES-SO), Lausanne, Vaud, Switzerland; 2 Karolinska Institutet, Stockholm, Stockholm Lans, Sweden; 3 Uiversity of Applied Sciences and Arts of Western Switzerland (HES-SO), Lausanne, Vaud, Switzerland; 4 Karolinska Institutet, Huddinge, Stockholms Lan, Sweden; 5 Karolinska Institute, Stockholm, Stockholms Lan, Sweden

## Abstract

For older adults to “age in place”, they need to keep engaged and mobile in their communities, whatever their health condition. The impact of age and cognitive decline on community mobility is a growing problem in Europe and worldwide. Engaging in occupations outside home implies being able to get to those places where activities are performed. Yet little is known regarding the types of places visited, maintained or abandoned for older adults with/without dementia. This study addresses community mobility needs through the places people visit, maintain or abandon. People with and without dementia, aged 55+, were interviewed using the Participation in ACTivities and Places OUTside the Home (ACT-OUT) questionnaire across Switzerland (n=70), Sweden (n=69) and the UK (n=128). Results show that people with dementia experience a higher rate of abandonment for more places than regular older adults. Insights about driving cessation and access to travel passes will be presented.

